# Radiological evaluation of response in patients with locally advanced/metastatic soft tissue sarcoma treated with trabectedin

**DOI:** 10.3389/fphar.2024.1411707

**Published:** 2024-08-20

**Authors:** S. Ceddia, C. E. Onesti, S. Vari, A. Torchia, A. Cosimati, F. Riva, M. T. Maccallini, M. Cerro, G. Benvenuti, M. Russillo, V. Anelli, I. Sperduti, R. Biagini, V. Ferraresi

**Affiliations:** ^1^ UOSD Sarcomas and Rare Tumors, IRCCS Regina Elena National Cancer Institute, Rome, Italy; ^2^ Scienze Radiologiche, Oncologiche e Anatomo-Patologiche, Sapienza Università di Roma, Rome, Italy; ^3^ UOC Oncologia Territoriale Ausl Latina, Aprilia, Italy; ^4^ Radiology, IRCCS Regina Elena National Cancer Institute, Rome, Italy; ^5^ Unit of Biostatistical, IRCCS Regina Elena National Cancer Institute, Rome, Italy; ^6^ Oncological Orthopaedics Unit, IRCCS Regina Elena National Cancer Institute, Rome, Italy

**Keywords:** sarcoma, soft tissue, trabectedin, response assessment, real-life

## Abstract

**Background:** Trabectedin is an antineoplastic drug approved for patients (pts) with advanced soft tissue sarcomas (STS). Interestingly, the radiological evaluation of response during trabectedin therapy is peculiar.

**Methods:** The aim of this single-center retrospective study is to analyze the concordance of response assessment according to RECIST compared with Choi criteria in patients with STS treated with trabectedin between 2009 and 2020 at Regina Elena National Cancer Institute in Rome.

**Results:** We present the preliminary data collected in the last 2 months (mos) on 37 pts who received the diagnosis between 2015 and 2020, with a median age of 52.5 years (range 32–78). The median number of trabectedin cycles administered was four (range 2–50) for a median follow up of 5.83 months (range 1–60). Histological subtypes of STS were five (13.5%) leiomyosarcoma, 14 (37.8%) liposarcoma, nine (24.3%) undifferentiated pleomorphic sarcoma, three (8.1%) synovial sarcoma, and six (16.2%) other rare histological subtypes. Eight pts (21.6%) received trabectedin in the first line setting, 21 (56.8%) in the second line, and seven (18.9%) received it in subsequent lines. One pt received trabectedin as neoadjuvant therapy in a clinical trial (ISG-STS 1001). Median progression-free survival was 3.6 months (CI95% 2.7–4.6); median overall survival was 34.3 months (CI95% 0–75.4). The radiological responses were evaluated with both RECIST and Choi criteria; responses matched in 33 pts (89.2%) but not in four (10.8%). The best responses obtained according to RECIST criteria were two (5.4%) partial response (PR), 13 (35.1%) stable disease (SD), and 22 (59.5%) progressive disease (PD). Instead, two (5.4%), 13 (35.1%), and 22 (59.5%) pts obtained PR, SD, and PD respectively, according to Choi criteria. Cohen’s kappa coefficient of concordance was 0.792 (*p*-value <0.002). A specialized radiologist performed all imaging examinations using a dedicated workstation in the same center.

**Conclusion:** In this first analysis, the concordance between RECIST and Choi assessments demonstrates no statistically significant difference. Responses did not match for four pts. We are expanding the analysis to all pts included in the original cohort to confirm or deny these initial results.

## 1 Introduction

Soft tissue sarcomas (STS) are rare tumors that arise from anatomical structures of mesenchymal origin. Their global incidence is around 3–5 cases/per 100,000 people/year, and they represent 1% of adult cancers (Sbaraglia et al., 2021). More than 50 different histological subtypes with specific biological characteristics and distinct behaviors are grouped under the term “STS”, and histological evaluation before any therapeutic step is mandatory to define the most correct therapeutic strategy. Magnetic resonance imaging (MRI) represents the main imaging modality for diagnosis and follow-up, especially for STS of the extremities, the pelvis, and the trunk. Computed tomography (CT) is another radiologic technique useful for staging and evaluation of response during active treatments and in follow up. PET is a second-choice test mainly used to better characterize CT or MRI findings or to identify bone metastases (Gronchi et al., 2021). Surgery is the cornerstone treatment for localized STS disease, followed by postoperative radiotherapy in high-grade (G2-3) lesions. Systemic treatment is based mainly on chemotherapy that can be offered in peri-operative settings (neo-adjuvant or adjuvant) for high-risk patients and in metastatic disease and anthracyclines alone or in combination with ifosfamide, and are the first line of reference for most chemosensitive histotypes (Gronchi et al., 2021; Blay et al., 2022). The second line is a limited number of chemotherapeutic agents proven to be active in STS. A greater understanding of the different chemosensitivities for each histological subtype with respect to different drugs has led to a histotype-tailored approach (Scurr, 2011). Among the drugs usually employed as second-line treatments, trabectedin was approved in 2007 by the European Medicines Agency (EMA) for use in patients with advanced STS after the failure of previous chemotherapies, including anthracyclines, or for patients not eligible for this latter treatment. Trabectedin is an anticancer drug; chemically it is a tetrahydroisoquinoline discovered in 1969 and is obtained from *Ecteinascidia turbinata*, a Caribbean Sea ascidian (Trabectedin: Ecteinascidin 743, 2006). This drug has proven to be particularly active in leiomyosarcomas and liposarcomas (especially myxoid liposarcomas), although responses were also obtained in other rarer histotypes (van Kesteren et al., 2003; Allavena et al., 2005; Vincenzi et al., 2010).

Trabectedin exhibits a complex mechanism of action by affecting key processes of cell biology at both the level of tumor cells and the tumor microenvironment. Unlike other alkylating agents that act on the major groove of DNA, trabectedin binds to the minor groove and interferes with DNA repair mechanisms, altering the transcription regulation of induced genes. This molecule also acts on the tumor microenvironment by modulating pro-tumor inflammatory phenomena through the induction of apoptosis of tumor-infiltrating macrophages (TAM) with associated reduced angiogenesis (D’Incalci and Jimeno, 2003; Germano et al., 2013).

The radiological evaluation of response during trabectedin chemotherapy is of particular interest. Preclinical studies have shown that trabectedin is effective in modulating the transcription of oncogenic fusion proteins, and clinically meaningful results were observed in sarcomas associated with translocations (e.g., myxoid liposarcoma and synovial sarcoma) (Scurr, 2011; Palmerini et al., 2022). Early in clinical development, trabectedin demonstrated relevant antitumor activity against myxoid-round cell liposarcoma (MRC-L-sarcoma). This high activity seems to be related to trabectedin’s ability to counteract the biological activity of the chimeric FUS-DDIT3 oncoprotein, a defining characteristic of this disease (Scurr, 2011; Palmerini et al., 2022). In myxoid liposarcoma, which is a type of sarcoma associated with specific translocation DDIT3-FUS or DDIT3-EWSR1, trabectedin proved to be particularly active, and a change in tumor density has often been observed, associated or not with a subsequent reduction in tumor diameters (Taieb et al., 2015; Baheti et al., 2017). Synovial sarcoma is characterized by the presence of the SS18-SSX fusion gene. Trabectedin can disrupt the transcriptional activity of the SS18-SSX fusion oncoprotein, inhibiting its role in cell proliferation and survival (Fiore et al., 2021).

Consequently, new “functional” imaging techniques have been proposed to assess treatment response that are capable of detecting tissue changes earlier before a change in size, such as MR perfusion (for qualitative-quantitative study vascularization and capillary permeability), MRI diffusion (to more accurately identify changes in cell density by quantifying the mobility of the water molecules present), and PET-TC (for the functional evaluation of the pathological tissues thanks to the variation of cellular metabolism) (Marcus et al., 2009; Baheti et al., 2017; Fanciullo et al., 2022). The Choi and the RECIST criteria are both used to assess tumor response to treatment, but they have different approaches. Choi criteria incorporates size and tumor attenuation (density) changes, while RECIST focuses solely on size changes (Choi et al., 2007; Eisenhauer et al., 2009).

The aim of this single-center retrospective observational cohort study is to evaluate patients with various histotypes of STS treated with trabectedin, comparing the traditional morphological criteria of response (response evaluation criteria in solid Tumors—RECIST) with “functional” criteria (Choi criteria) (Taieb et al., 2015).

## 2 Patients and methods

Eligible patients were adults (age ≥18 years) with various histotypes of STS undergoing treatment with trabectedin after a confirmed local relapse or metastatic disease. Other main inclusion criteria were: Eastern Cooperative Oncology Group performance status (ECOG PS) ≤ 2; normal bone marrow, liver and kidney function; availability of CT for the assessments under study; availability of clinical follow-up. The study has been conducted under the principles of “Good Clinical Practice” required by the regulatory authorities and the main European and national regulations. The data, material and documentation related to the study were collected, stored, and processed following the provisions of the relevant legislation/regulations in a manner that guarantees its confidentiality. The study was conducted in accordance with the Declaration of Helsinki and current legislation in this regard and has been approved by the local ethics committee. Written informed consent from the participants was not required following national legislation and institutional requirements.

### 2.1 Study design and endpoints of the study

This is a single-center retrospective observational cohort study on patients with STS who are undergoing treatment with trabectedin at the Regina Elena National Cancer Institute in Rome (European Reference Network for Rare Adult Solid Cancers—EURACAN—referral center) over the reference time 2015–2020. The aim of this study was to evaluate radiological best response as assessed by CT scan in patients with unselected histotypes of STS treated with trabectedin, comparing the traditional morphological criteria of response (RECIST) with “functional” radiological evaluation criteria (Choi criteria). As per clinical practice, re-evaluation with CT was performed every three courses of treatment or at any time when disease progression was clinically suspected. Response assessment to decide continuation (disease response or stabilization) or discontinuation (disease progression) of trabectedin therapy was performed according to RECIST criteria.

### 2.2 Statistical analyses

From 2015 to 2020, the data relating to all the patients who meet the envisaged requirements were analyzed and processed. Descriptive statistics were calculated for all variables of interest. Categorical variables were reported through absolute frequencies and relative percentage values, while continuous variables will be reported through medians and ranges. All associations among the categorical variables considered were evaluated by Pearson’s chi-square test or Fisher’s exact test. DFS and OS curves were evaluated by the Kaplan–Meier method and the Mantel–Haenszel log-rank test, which were employed to compare survival between groups. Hazard ratio (HR) and odds ratio (OR) estimates, which allow quantification of the relative effect of each predictor on the outcome considered, and the corresponding 95% confidence intervals were calculated using the Cox regression model with proportional hazards and the logistic regression model. A *p*-value ≤0.05 was considered statistically significant.

## 3 Results

We present data collected on 37 patients (pts) who received the diagnosis over 2015–2020, with a median age of 52.5 years (range 32–78) (Table 1).

**TABLE 1:** General demographic and clinical characteristics in treated patients.

Treated pts, n (%)	37	100%
Median age, years (range)	**52.5 (32–78)**	
Gender, M/F	**21/16**	
Histological subtypes, n (%)	**37**	**100%**
• Liposarcoma	14	38%
• Undifferentiated pleomorphic sarcoma	9	24%
• Leiomyosarcoma	5	14%
• Synovial sarcoma	3	8%
• Other	6	16%
Sarcoma primitive lesion, n (%)	**37**	**100%**
• Extremities	23	62%
• Retroperitoneal	8	22%
• Trunk	6	16%
Stage of disease at diagnosis, n (%)	**37**	**100%**
• Locally advanced	10	27%
• Metastatic	27	73%
Previous treatments, n (%)		
• Surgery	34	92%
• Radiotherapy	5	14%
• Chemotherapy	32	86%
Median number of previous metastatic systemic treatments, n (range)	**3 (1–5)**	
Starting dose of trabectedin, n (%)	**37**	**100%**
• 1.3 mg m2	12	32%
• 1.5 mg/mq	1	3%
• n.a.	24	65%
Median duration of treatment with trabectedin, months (range)	**5.8 (1–60)**	
Median number of trabectedin cycles, n (range)	**4 (1–60)**	
Line of therapy with trabectedin, n (%)	**37**	**100%**
• First line	8	22%
• Second line	22	59%
• Subsequent lines	7	19%

Abbreviation: n.a., not applicable. The bold value indicates the total number of patients for each main section.

**FIGURE 1:**
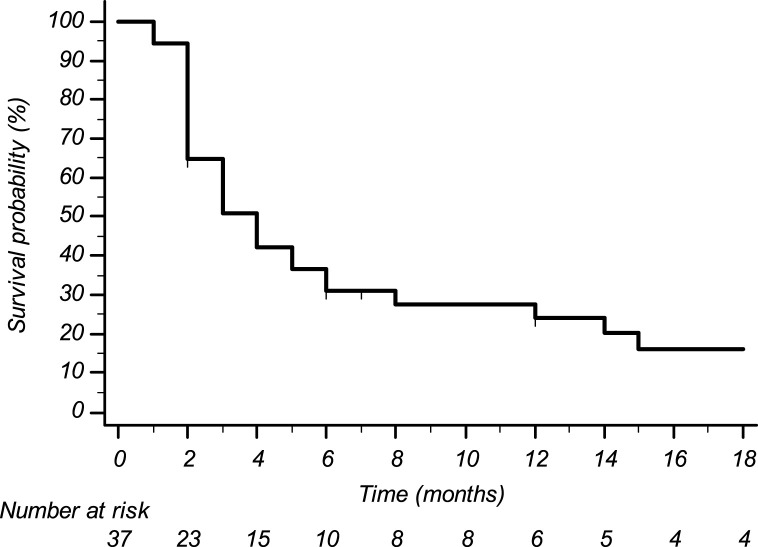
Progression-free survival.

**FIGURE 2:**
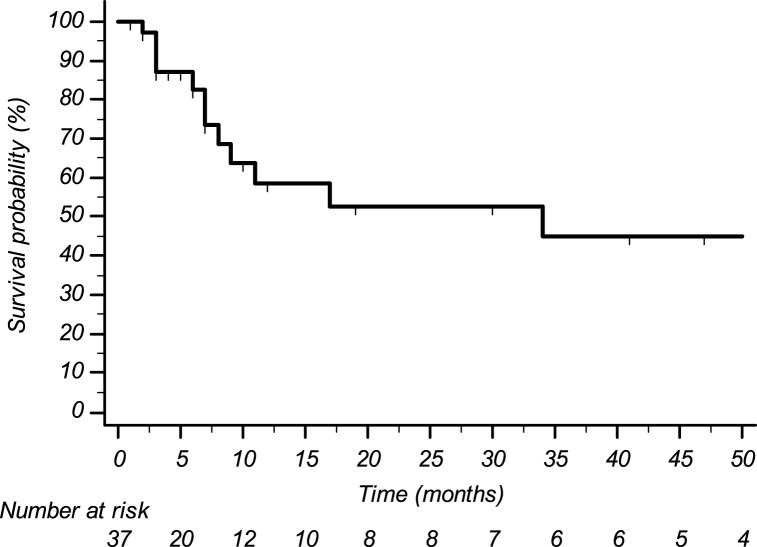
Overall survival.

Histological subtypes of STS were five (13.5%) leiomyosarcoma, 14 (37.8%) liposarcoma, nine (24.3%) undifferentiated pleomorphic sarcoma, three (8.1%) synovial sarcoma, and six (16.2%) other histological subtypes. Eight pts (21.6%) received trabectedin in the first-line setting (five had previously undergone treatment with anthracyclines in the adjuvant or neoadjuvant setting; three had contraindication to anthracyclines due to cardiac comorbidities), 22 pts (59.5%) in the second line (of whom 20 were treated with anthracyclines +/- ifosfamide in the neoadjuvant, adjuvant, or first-line setting, and two were treated with anthracyclines +/- ifosfamide in the neoadjuvant or adjuvant setting and subsequently received gemcitabine-docetaxel), and seven pts (18.9%) in subsequent lines. The median number of administered trabectedin cycles was four (range 2–50) with a median treatment duration of 5.8 months (range 1–60). Median progression-free survival was 3.6 months (CI95% 2.7–4.6) (Figure 1); median overall survival was 34.3 months (CI95% 0–75.4) (Figure 2).

A specialized radiologist performed all the imaging examinations using a dedicated workstation in the same center. The radiological responses were evaluated with both RECIST and, retrospectively, Choi criteria. The best responses obtained according to RECIST criteria were two (5.4%) partial response (PR) represented by a pleomorphic liposarcoma (PLPS) and an undifferentiated pleomorphic sarcoma (UPS), 13 (35.1%) stable disease (SD), and 22 (59.5%) progressive disease (PD). Two (5.4%), 13 (35.1%), and 22 (59.5%) pts obtained PR, SD and PD respectively according to CHOI criteria (Table 2).

**TABLE 2:** Best response according to physician evaluation RECIST, Choi N, %.

All treated pts, N = 37	RECIST criteria	Choi criteria
Partial response (PR)	2 (5.4%)	2 (5.4%)
Stable disease (SD)	13 (35.1%)	13 (35.1%)
Progressive disease (PD)	22 (59.5%)	22 (59.5%)

In 33 pts (89.2%), the responses assessed according to RECIST and Choi criteria matched, whereas four pts (10.8%) did not match. Two pts were considered in SD according to RECIST 1.1 and PD with Choi criteria; two others with PD according to RECIST 1.1 were classified as SD with Choi criteria (Table 3). In pts 1 and 4, PD according to CHOI criteria was represented by an increase in the vascularized intralesional component, while dimensional stability was observed as per RECIST criteria. In pts 2 and 3, PD is attributed to an increase in the size of the target lesions, while SD was observed according to CHOI criteria due to intralesional remodeling and an increase in tissue density.

**TABLE 3:** Patients with RECIST and Choi criteria dissociated responses.

Patients, N = 4	Histological subtypes	RECIST criteria	Choi criteria
Patient 1	Pleomorphic liposarcoma	SD	PD
Patient 2	Leiomyosarcoma	PD	SD
Patient 3	Alveolar sarcoma	PD	SD
Patient 4	Myxoid liposarcoma	SD	PD

Cohen’s kappa coefficient of concordance was 0.792 (*p*-value <0.002). The first pt affected by liposarcoma showed SD according to RECIST criteria and PD according to CHOI criteria. She discontinued trabectedin treatment and is reported as lost to follow-up. Pt 2, diagnosed with leiomyosarcoma, underwent trabectedin treatment in the second line and subsequently, following RECIST-defined disease progression, received three additional lines of therapy with modest benefit (gemcitabine-docetaxel, dacarbazine, and ifosfamide with PD after three, five and two cycles of treatment, respectively). Pt 3, with alveolar sarcoma, initiated trabectedin treatment in the sixth line and maintained disease stability for 21 months. Later, the pt underwent another and final line of treatment with off-label bevacizumab, with rapid disease progression after 3 months. Pt 4 underwent surgery after showing disease stability according to RECIST criteria, followed by a disease-free interval of 2 years. The treatment with trabectedin was overall well-tolerated. The most frequently reported toxicities were neutropenia and transient transaminase increase according to the literature. All pts received steroid pre- and post-medication as per recommended dosage.

## 4 Discussion

The RECIST 1.1 guidelines (Eisenhauer et al., 2009) represent the system mainly used for the assessment of disease status based on changes in tumor size. In selected cases, such as during treatment with tyrosine kinase inhibitors, different assessment methods could be useful because both changes in volume and density may better represent drug activity instead of classical two dimensional evaluation (Schuetze, 2005). Treatment-related changes in STS, especially assessment of trabectedin response, have been shown to be closely related to altered tumor composition and density; thus therapeutic benefit without tumor shrinkage appears to be relevant in STS (Schuetze, 2005). This novel response pattern was first described by Choi et al. (2007), defining it in the setting of patients with gastrointestinal stromal tumor (GIST) treated with imatinib. They described criteria based on both dimensional and density changes in GIST treated with the TKI imatinib, arguing that RECIST criteria significantly underestimate tumor response. Specifically, variations in tumor mass dimensions may not accurately reflect tumor activity; changes in tumor density represent an additional measure of treatment response, which can be objectively assessed and measured based on radiological images (Choi et al., 2007). In a retrospective study, Taieb et al. (2015) suggested that Choi’s criteria can help identify cases of false progression (tumor progression according to RECIST but PR or SD according to CHOI criteria), demonstrating a longer OS in those patients compared to cases where progression is confirmed by both RECIST and Choi criteria. In this patient setting, the correct definition of disease progression is therefore crucial, considering the decisions in therapeutic strategies and the impact on disease outcomes (Taieb et al., 2015). Dependent on histology and treatment, different changes can be distinguished after therapy such as nectrotic cells, granulation tissue, fibrosis, and calcifications (Lucas et al., 2008). Edema and intratumoral hemorrhage may show radiological changes in terms of increase in size, despite an excellent histologic response. In this case, a stability or progression of disease according to RECIST criteria could underestimate a histopathology (Lucas et al., 2008). Different radiological techniques such as MRI, CT, and 18F-FDG PET could detect these changes in order to formulate a more appropriate definition of radiological responses (Gennaro et al., 2021).

In our real life analysis, the overall disease control rate was 40%, consistent with recent retrospective analyses (Palmerini et al., 2021). The lower objective response rate (only 5%) with a median PFS of 3.6 months could be explained by both the small sample size of our patient population and the previous lines of treatment received by about 20% of patients on their third and greater line of systemic therapy. Radiological evaluation with Choi criteria changed the response in four cases. One of the two patients in SD according to CHOI criteria had alveolar soft part sarcoma. A Choi criteria evaluation would have allowed the patient to be maintained on therapy, especially considering the limited therapeutic options in this histotype and in a patient with highly pretreated disease. Additionally, alveolar soft part sarcoma has proven to be a histotype that is responsive to trabectedin as per Taieb et al. (2015). The other patient, after disease progression according to RECIST criteria, exhibited a brief response to subsequent treatments (short PFS). Conversely, an assessment of treatment response according to Choi functional criteria would have allowed the patient to continue trabectedin treatment. The first patient was an elderly woman affected by bulky abdominal disease with bone involvement, and she is reported as lost to follow-up; therefore, it is challenging to understand whether the early detection of progression with Choi criteria may have impacted the prognosis. The patient diagnosed with myxoid liposarcoma underwent surgery after disease stability according to RECIST criteria and experienced a recurrence of the disease 2 years after the trabectedin treatment. It is plausible that the limited progression detected by Choi criteria could have provided a positive impact on the prognosis of this pt in the absence of further systemic treatments.

The differences between the two assessment strategies did not show statistical significance, as Cohen’s coefficient of agreement kappa was 0.792 (*p*-value <0.002). Use of the standard evaluation executed with both RECIST and Choi criteria remains a challenge as it requires a specialist radiologist for this patient setting. Indeed, it is internationally recognized that the rarity of sarcomas and the variety of histotypes imply that a multidisciplinary approach, including a radiological evaluation of the response by a dedicated and experienced radiologist, in a referral center remains the most effective way to impact the prognosis of these malignancies (Gronchi et al., 2021).

This study represents a first analysis, and secondary objectives are underway for identifying, in patients affected by STS treated with trabectedin, predictive or prognostic parameters according to objective response, progression-free survival (PFS), and overall survival (OS). There are some limitations due to the limited number of cases and, consequently, less power in the comparison of radiological tumor response assessments. Furthermore, the study had a retrospective design. A larger cohort and a prospective multicenter study would be necessary to achieve more consistent results.

## 5 Conclusion

A functional assessment combined with changes in tumor size is crucial in patients with advanced STS treated with trabectedin in order to prevent an early treatment interruption that may deprive the patient of a therapeutic option. In this first analysis, the concordance between RECIST and CHOI assessments demonstrates no statistically significant difference. Responses did not match for four patients. The goal is to define a consistent and unbiased evaluation of the efficacy of both local and systemic therapies through imaging to find more personalized therapeutic approaches.

## Data Availability

The original contributions presented in the study are included in the article/Supplementary Material; further inquiries can be directed to the corresponding author.
